# Impact of Neoadjuvant Androgen Deprivation Therapy on Urinary Continence and Longitudinal Quality of Life After Robot-Assisted Radical Prostatectomy

**DOI:** 10.5152/tud.2026.26026

**Published:** 2026-08-19

**Authors:** Hisanori Taniguchi, Junichi Ikeda, Yuki Masuo, Hidefumi Kinoshita

**Affiliations:** Department of Urology and Andrology, Kansai Medical University, Hirakata, Osaka, Japan

**Keywords:** Robot-assisted radical prostatectomy, neoadjuvant androgen deprivation therapy, urinary continence, health-related quality of life

## Abstract

**Objective:**

Neoadjuvant androgen deprivation therapy (ADT) is commonly administered before robot-assisted radical prostatectomy (RARP) in selected patients with localized prostate cancer, particularly those with high-risk disease. Despite its potential oncologic benefits, the association of neoadjuvant ADT with postoperative urinary function and health-related quality of life remains unclear. Therefore, this study aims to evaluate the association between neoadjuvant ADT and functional recovery following RARP.

**Methods:**

Consecutive patients who underwent RARP between January 1, 2022, and December 31, 2024, were retrospectively reviewed and categorized based on neoadjuvant ADT status. Urinary continence (≤1 pad/d), Expanded Prostate Cancer Index Composite urinary domain score, Functional Assessment of Cancer Therapy—General score, and Functional Assessment of Cancer Therapy—Prostate score were assessed longitudinally. Urinary continence recovery was analyzed using the Kaplan–Meier method, and longitudinal patient-reported outcomes were evaluated using linear mixed-effects models.

**Results:**

Among 327 eligible patients, 58 received neoadjuvant ADT. Urinary continence recovery was comparable between the groups (log-rank *P* = .251). Expanded Prostate Cancer Index Composite urinary domain scores decreased immediately after surgery and improved over time (*P* for time <.001). Neoadjuvant ADT (*P* = .181) and the time-by-treatment interaction (*P* = .557) were not significantly associated with postoperative urinary function. Similarly, longitudinal Functional Assessment of Cancer Therapy—General and Functional Assessment of Cancer Therapy—Prostate scores were comparable between the groups.

**Conclusion:**

Neoadjuvant ADT is not associated with delayed urinary continence recovery or poorer health-related quality of life after RARP, supporting its use in appropriately selected patients without evidence of compromised postoperative functional recovery.

**Main Points**

- Neoadjuvant androgen deprivation therapy (ADT) before robot-assisted radical prostatectomy was not associated with delayed postoperative urinary continence recovery.
- Longitudinal patient-reported outcomes demonstrated similar urinary function trajectories in patients with and without neoadjuvant ADT.
- Overall and prostate cancer-specific health-related quality of life followed similar recovery trajectories regardless of neoadjuvant ADT status.
- These findings support the use of neoadjuvant ADT before robot-assisted radical prostatectomy in appropriately selected patients without compromising postoperative functional recovery or health-related quality of life.

## Introduction

Robot-assisted radical prostatectomy (RARP), a standard treatment for localized prostate cancer, offers excellent oncologic control while preserving postoperative urinary and functional outcomes. In patients with high-risk localized prostate cancer, neoadjuvant androgen deprivation therapy (ADT) is sometimes administered before surgery to reduce tumor burden and improve pathological outcomes. Studies report favorable effects of neoadjuvant ADT on pathological downstaging and surgical margin status; however, its effect on postoperative functional recovery remains uncertain.^1-3^

Prospective studies consistently show that ADT reduces lean body mass and skeletal muscle strength while increasing the risk of sarcopenia, potentially impairing physical performance and postoperative recovery.^4,5^ Pelvic floor muscle function is a key determinant of urinary continence after radical prostatectomy; therefore, ADT-induced muscle loss may adversely affect postoperative urinary function and continence recovery.^6^ Despite these concerns, clinical evidence on the functional effects of neoadjuvant ADT before RARP remains limited. Most studies have focused on perioperative safety, pathological outcomes, and oncologic outcomes, whereas longitudinal assessments of patient-reported urinary function and health-related quality of life (HR-QOL) have received less attention. Furthermore, studies concurrently evaluating urinary continence recovery and validating patient-reported outcome measures after RARP remain limited.

Therefore, this study aims to investigate the association between neoadjuvant ADT and postoperative urinary continence recovery, urinary function, and longitudinal HR-QOL outcomes following RARP.

## Material and Methods

### Study Population

This single-center retrospective cohort study included consecutive patients who underwent standard transabdominal RARP between January 1, 2022, and December 31, 2024. Only patients with postoperative urinary continence assessment data were included in the analysis. Patients were classified based on preoperative neoadjuvant ADT use. Neoadjuvant ADT comprised a luteinizing hormone-releasing hormone agonist or antagonist, with or without an antiandrogen, administered before surgery.

The institutional ethics committee approved this retrospective study on October 3, 2022 (Approval no. 2022137). The approved protocol authorized a retrospective review of existing medical records of patients treated during the predefined study period (January 1, 2022, to December 31, 2024), including those who underwent surgery before ethics approval. Accordingly, all patients included in this study were covered by the approved retrospective protocol. This study was conducted following the principles of the Declaration of Helsinki. The requirement for informed consent was waived due to the retrospective study design; however, participants were informed about the study and given the opportunity to withdraw.

### Outcome Measures

The primary outcome was urinary continence recovery following RARP. Urinary continence was defined as the use of ≤1 pad/d and was evaluated using Question 5 of the Expanded Prostate Cancer Index Composite (EPIC) questionnaire: “*How many pads or adult diapers per day did you usually use to control leakage during the last 4 weeks?*”^7^ Secondary outcomes included longitudinal changes in urinary function, assessed using the EPIC urinary domain, and HR-QOL, assessed with the Functional Assessment of Cancer Therapy—General (FACT-G) and Functional Assessment of Cancer Therapy—Prostate (FACT-P).^8^

### Statistical Analysis

Patient characteristics were summarized using descriptive statistics. Continuous variables were compared using the Mann–Whitney *U* test or Student’s *t* test, as appropriate based on data distribution, while categorical variables were compared using the chi-square test or Fisher’s exact test.

Time to urinary continence recovery was estimated using the Kaplan–Meier method, and group differences were assessed with the log-rank test. Longitudinal changes in EPIC and FACT scores were analyzed using linear mixed-effects models with fixed effects for time, neoadjuvant ADT status, and the time-by-treatment interaction, along with a participant-specific random intercept to account for repeated measurements.

All statistical analyses were performed using IBM SPSS Statistics for Windows, version 28.0 (IBM Corp., Armonk, NY, USA). Statistical significance was defined as a 2-sided *P* < .05. This study used ChatGPT (OpenAI, San Francisco, CA, USA) during manuscript preparation to improve the readability and quality of English expression. Following the use of this AI/LLM tool, all scientific content was critically reviewed and edited by the authors, and the final manuscript underwent professional English language editing.

## Results

### Patient Baseline Characteristics

Table 1 presents the patient baseline characteristics. The study cohort comprised 327 patients, including 58 who received neoadjuvant ADT. Indications for neoadjuvant ADT were high-risk disease (*n* = 42), disease control during the surgical waiting period (*n* = 12), and prostate downsizing before RARP (*n* = 3). The median duration of neoadjuvant ADT was 6 months. Patients receiving neoadjuvant ADT exhibited higher-risk disease characteristics, including higher prostate-specific antigen levels and lower rates of nerve-sparing procedures.

**Table 1.** Baseline Characteristics of the Study Cohort

Variable	ADT (−) (*n* = 279)	ADT (+) (*n* = 58)	*P* Value
Age, years	69.1 ± 6.4	68.7 ± 5.6	.697
BMI, kg/m^2^	23.6 ± 3.1	23.8 ± 3.0	.545
≥cT3, *n* (%)	2 (0.7)	10 (17)	<.001
PSA, ng/mL	8.49 ± 5.33	23.3 ± 27.2	<.001
Biopsy GG ≥ 4, *n* (%)	90 (32)	48 (83)	<.001
High risk, *n* (%)	96 (34.4)	50 (86.2)	<.001
PLND, n (%)	116 (42)	46 (80)	<.001
Nerve sparing, *n* (%)	212 (76)	23 (40)	<.001
G8 score	14.7 ± 1.55	14.5 ± 1.50	.271
Duration of ADT, months	—	7.0 ± 5.5	—

Values are presented as mean ± SD or number (%), and PSA was compared using the Mann–Whitney *U* test. Other continuous variables were compared using Student’s *t* test.

ADT, androgen deprivation therapy; BMI, body mass index; GG, Gleason grade; PLND, pelvic lymph node dissection; PSA, prostate-specific antigen; SD, standard deviation.

### Pathological and Oncologic Outcomes Based on Neoadjuvant ADT Status

Table 2 presents surgical procedures and pathological and oncologic outcomes in the neoadjuvant ADT and non-ADT groups. The neoadjuvant ADT group had a lower specimen weight and a higher final pathological grade than the non-ADT group. During the follow-up, biochemical recurrence incidence was similar between the groups.

**Table 2.** Pathologic and Oncologic Outcomes Based on Neoadjuvant ADT Status Before Robot-Assisted Radical Prostatectomy

Variable	ADT (−) (*n* = 279)	ADT (+) (*n* = 58)	*P* Value
Surgical procedure			
Console time (min), mean ± SD	197.3 ± 43.7	210.8 ± 46.2	.053
Estimated blood loss (mL), mean ± SD	338.9 ± 272.3	334.1 ± 329.6	.681
Specimen weight (g), mean ± SD	42.1 ± 16.9	35.3 ± 20.6	<.001
Pathological outcomes			
Extraprostatic extension, *n* (%)	46 (16.5)	15 (25.9)	.095
Seminal vesicle invasion, *n* (%)	12 (4.3)	8 (13.8)	.011
Pelvic lymph node metastasis (pN+), *n* (%)	5/116 (4.3)	3/46 (6.5)	.558
Positive surgical margin, *n* (%)	40 (14.3)	4 (6.9)	.140
Final pathological grade group ≥ 4, *n* (%)	64 (22.9)	32 (55.2)	<.001
Oncological outcomes			
Follow-up duration, months, median (range)	23 (4-42)	29 (2-42)	.317
Biochemical recurrence, *n* (%)	41 (14.7)	9 (15.5)	.873

ADT, androgen deprivation therapy; SD, standard deviation.

### Postoperative Urinary Function Outcomes

Figure 1 shows urinary continence status (≤1 pad/d) after RARP. No significant difference in continence recovery was observed between patients with and without neoadjuvant ADT (*P* = .251). Continence rates at 6 and 12 months were 93% and 96%, respectively, in the non-ADT group and 82% and 92%, respectively, in the neoadjuvant ADT group. EPIC urinary domain scores significantly decreased immediately after surgery and gradually improved over time (*P* < .001). Neoadjuvant ADT was not significantly associated with overall EPIC urinary domain scores (*P* = .181), and the time-by-treatment interaction was not significant (*P* = .557; Figure 2).

**Figure 1.**
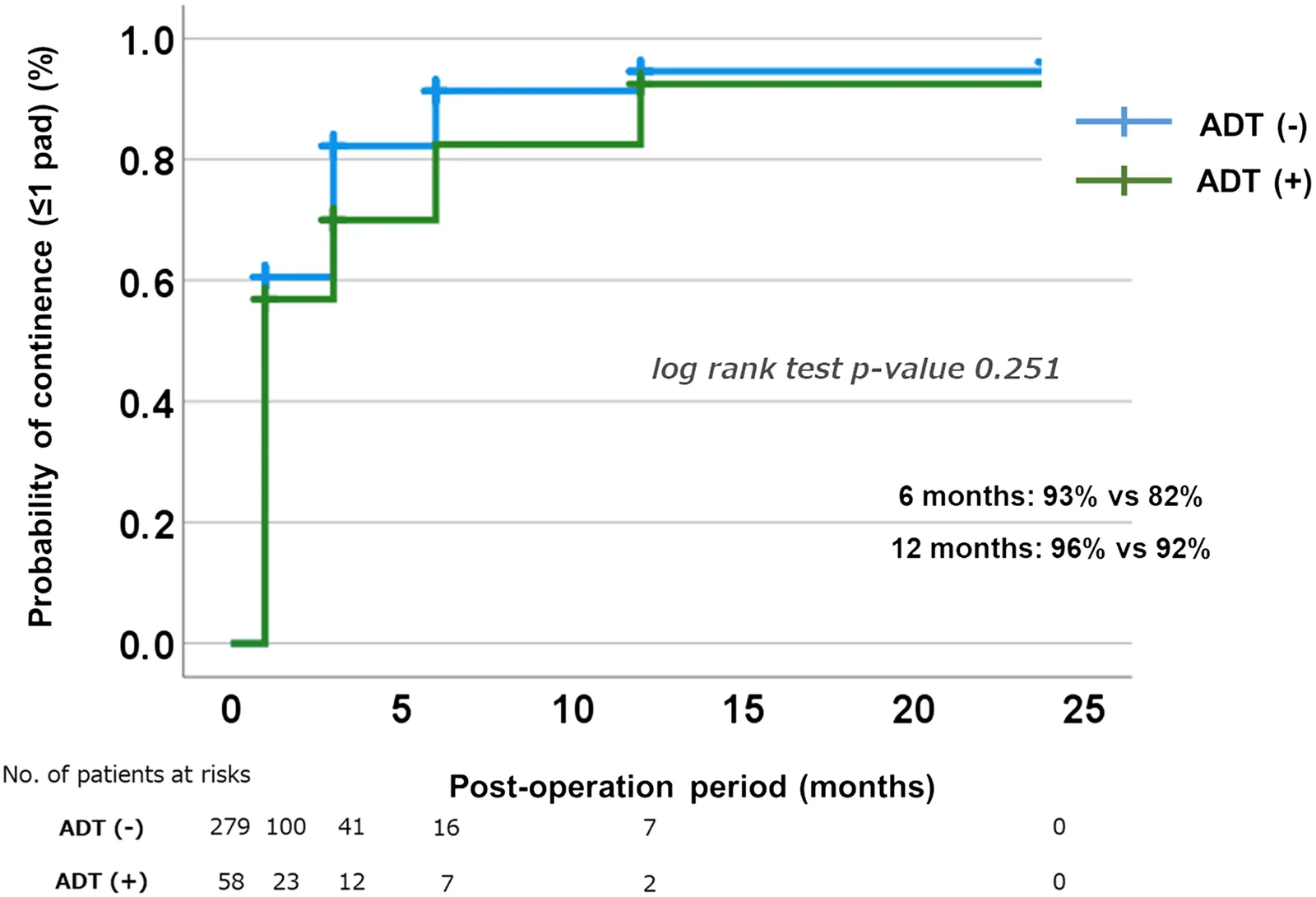
Kaplan–Meier curves showing time to urinary continence recovery (≤1 pad/d) after robot-assisted radical prostatectomy. Urinary continence was defined as the first postoperative assessment with patients reporting the use of ≤1 pad/d. Group differences were evaluated using the log-rank test.

**Figure 2.**
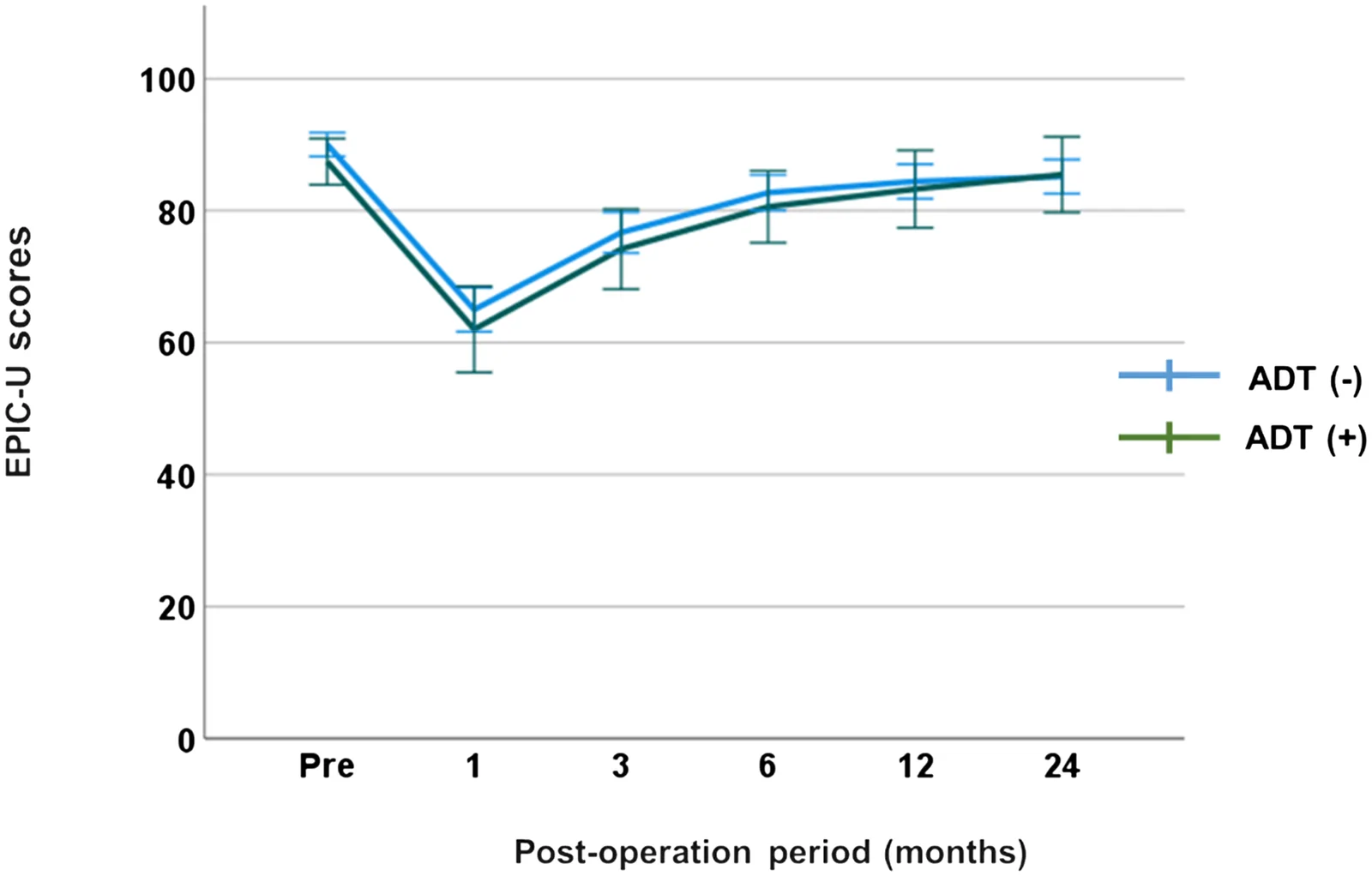
Longitudinal changes in EPIC urinary domain scores based on neoadjuvant ADT status. Values are presented as estimated marginal means with 95% confidence intervals derived from a linear mixed-effects model including fixed effects for time, neoadjuvant ADT status, and the time-by-treatment interaction, along with a patient-specific random intercept. *P* < .001 for time, *P* = .181 for neoadjuvant ADT, and *P* = .557 for the time-by-treatment interaction. ADT, androgen deprivation therapy; EPIC, Expanded Prostate Cancer Index Composite.

### Longitudinal HR-QOL Outcomes

Longitudinal changes in HR-QOL, evaluated using the FACT-G and FACT-P instruments, were similar between the 2 groups (Figure 3). FACT-G scores were not significantly associated with neoadjuvant ADT status (*P* = .938), and the time-by-treatment interaction was not significant (*P* = .642). Similarly, FACT-P scores were not significantly associated with neoadjuvant ADT (*P* = .962), and the time-by-treatment interaction was not significant (*P* = .993). Overall, longitudinal changes in general and prostate cancer-specific HR-QOL were comparable between the 2 groups. These findings indicate that neoadjuvant ADT does not adversely affect postoperative urinary function or HR-QOL following RARP.

**Figure 3.**
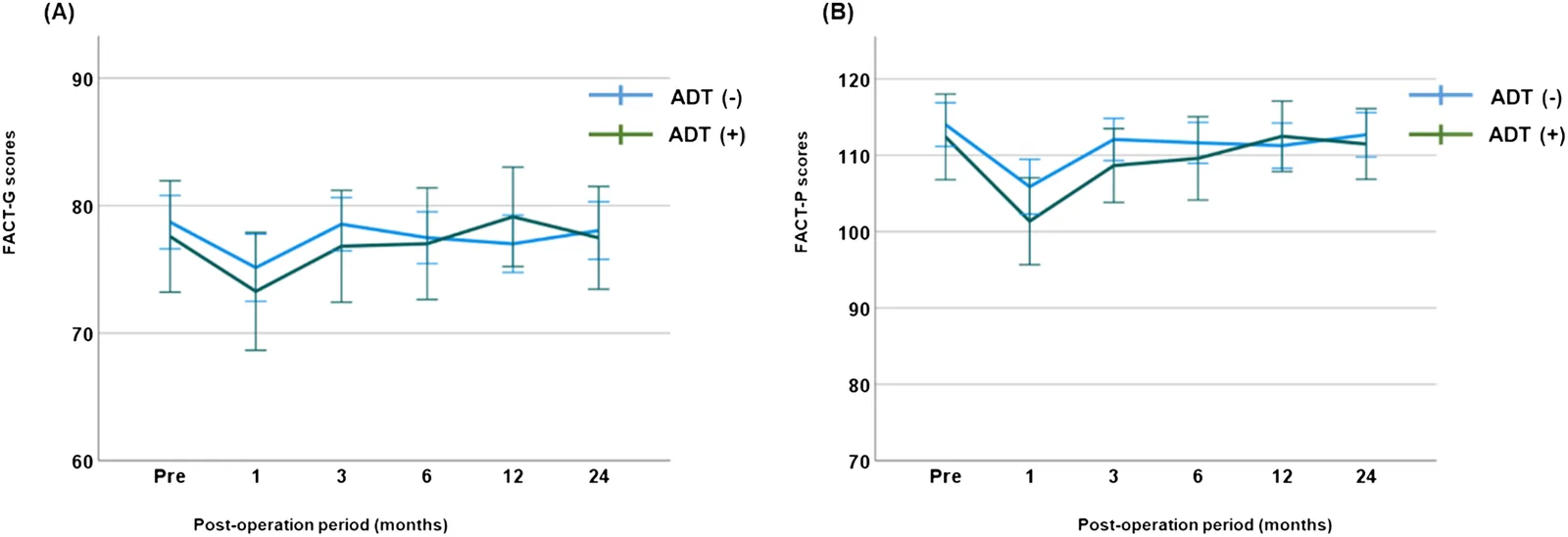
Longitudinal changes in health-related quality of life based on neoadjuvant ADT status. Longitudinal changes in health-related quality of life after robot-assisted radical prostatectomy were assessed using (A) FACT-G and (B) FACT-P. Values are presented as estimated marginal means with 95% CIs derived from linear mixed-effects models including fixed effects for time, neoadjuvant ADT status, and the time-by-treatment interaction, along with a patient-specific random intercept. ADT, androgen deprivation therapy; CI, confidence interval; FACT-G, Functional Assessment of Cancer Therapy—General; FACT-P, Functional Assessment of Cancer Therapy—Prostate.

## Discussion

Neoadjuvant ADT was not associated with impaired urinary continence recovery or HR-QOL after RARP in a contemporary real-world cohort. Time to urinary continence recovery did not differ significantly between patients with and without neoadjuvant ADT. Longitudinal linear mixed-effects analyses revealed no significant effect of neoadjuvant ADT on EPIC urinary domain scores or HR-QOL assessed using the FACT-G and FACT-P instruments.

Studies examining neoadjuvant ADT in patients undergoing RARP have primarily focused on perioperative and oncologic outcomes. Farzat et al reported no increase in major complications or 90-day readmissions among patients with neoadjuvant ADT before RARP.^2^ Similarly, Sangkum et al demonstrated that neoadjuvant ADT is associated with a shorter operative time, reduced estimated blood loss, and lower specimen weight, while surgical margin status and adjacent organ injury rates were similar between groups.^9^ Liu et al reported that neoadjuvant ADT in patients with high-risk prostate cancer significantly reduces lymph node involvement (risk ratio [RR] = 0.69; 95% confidence interval [CI]: 0.56-0.87) and increases the rates of pathological downstaging (RR = 2.62; 95% CI: 1.22-5.61) and organ-confined disease (RR = 2.24; 95% CI: 1.54-3.25).^3^ While the effect of neoadjuvant ADT on overall survival remains controversial,^3,10^ it may be considered as a treatment option for selected patients with high-risk localized prostate cancer undergoing RARP.

Studies examining the effect of neoadjuvant ADT on urinary function remain limited. Sun et al reported faster recovery of urinary continence after laparoscopic prostatectomy in patients receiving neoadjuvant ADT than in the control group^11^; however, this effect is less evident in the RARP cohort.^11^ In this study, neoadjuvant ADT was not associated with delayed urinary continence recovery or poorer patient-reported urinary function following RARP. Longitudinal mixed-effects modeling of validated patient-reported outcome measures showed similar continence recovery trajectories and EPIC urinary domain scores in patients receiving ADT and those who did not.

Several factors may account for the absence of an association between neoadjuvant ADT and impaired urinary continence recovery or poorer HR-QOL in this RARP cohort. First, contemporary robot-assisted surgery enables precise dissection and reconstruction, potentially mitigating the adverse effects of preoperative systemic therapy on postoperative functional recovery. Second, neoadjuvant ADT was administered for a limited duration. While ADT induces endocrine and musculoskeletal changes, including loss of lean body mass, these effects may be partially reversible after treatment discontinuation and likely insufficient to produce a sustained effect on postoperative urinary function.^4,5^ Third, functional recovery after RARP is highly time dependent, and progressive postoperative improvement may outweigh transient preoperative physiological changes.

Some limitations should be acknowledged. First, the retrospective design may have introduced selection bias because neoadjuvant ADT was more frequently administered to patients with higher-risk disease. Second, neoadjuvant ADT duration and treatment regimens were not standardized across patients, which may have influenced functional outcomes. Third, the relatively short follow-up period limited long-term oncologic outcome assessment, including biochemical recurrence. Despite these limitations, urinary function and HR-QOL recovery trajectories were evaluated comprehensively. These findings indicate that neoadjuvant ADT does not impair postoperative functional recovery or HR-QOL even when selectively administered to patients with higher-risk disease.

In conclusion, neoadjuvant ADT is not associated with delayed urinary continence recovery or poorer longitudinal HR-QOL following RARP. These findings suggest that neoadjuvant ADT before RARP does not compromise postoperative functional recovery or HR-QOL and may be considered for appropriately selected patients without significant concern for adverse functional outcomes.

## Data Availability

The data that support the findings of this study are available on request from the corresponding author.
